# Dietary restriction abrogates antibody production induced by a DNA vaccine encoding the mycobacterial 65 kDa heat shock protein

**DOI:** 10.1186/1479-0556-7-11

**Published:** 2009-07-16

**Authors:** Larissa Lumi Watanabe Ishikawa, Thaís Graziela Donegá França, Fernanda Chiuso-Minicucci, Sofia Fernanda Gonçalves Zorzella-Pezavento, Nelson Mendes Marra, Paulo Câmara Marques Pereira, Célio Lopes Silva, Alexandrina Sartori

**Affiliations:** 1 Department of Microbiology and Immunology, Biosciences Institute, São Paulo State University (UNESP), Botucatu, São Paulo, 18618-000, Brazil; 2 Department of Parasitology, Biosciences Institute, São Paulo State University (UNESP), Botucatu, São Paulo, 18618-000, Brazil; 3 Department of Tropical Diseases, Medical School, São Paulo State University (UNESP), Botucatu, São Paulo, 18618-000, Brazil; 4 Department of Biochemistry and Immunology, University of São Paulo (USP), Ribeirão Preto, São Paulo, 14049-900, Brazil

## Abstract

**Background:**

Protein-calorie malnutrition (PCM) is the most common type of malnutrition. PCM leads to immunodeficiency and consequent increased susceptibility to infectious agents. In addition, responses to prophylactic vaccines depend on nutritional status. This study aims to evaluate the ability of undernourished mice to mount an immune response to a genetic vaccine (pVAXhsp65) against tuberculosis, containing the gene coding for the heat shock protein 65 from mycobacteria.

**Methods:**

Young adult female BALB/c mice were fed *ad libitum *or with 80% of the amount of food consumed by a normal diet group. We initially characterized a mice model of dietary restriction by determining body and spleen weights, hematological parameters and histopathological changes in lymphoid organs. The ability of splenic cells to produce IFN-gamma and IL-4 upon *in vitro *stimulation with LPS or *S. aureus *and the serum titer of specific IgG1 and IgG2a anti-hsp65 antibodies after intramuscular immunization with pVAXhsp65 was then tested.

**Results:**

Dietary restriction significantly decreased body and spleen weights and also the total lymphocyte count in blood. This restriction also determined a striking atrophy in lymphoid organs as spleen, thymus and lymphoid tissue associated with the small intestine. Specific antibodies were not detected in mice submitted to dietary restriction whereas the well nourished animals produced significant levels of both, IgG1 and IgG2a anti-hsp65.

**Conclusion:**

20% restriction in food intake deeply compromised humoral immunity induced by a genetic vaccine, alerting, therefore, for the relevance of the nutritional condition in vaccination programs based on these kinds of constructs.

## Background

Protein-calorie malnutrition (PCM) is still the most common type of undernutrition and approximately 800 million people in the world present some kind of malnutrition [1]. This deficiency is usually complex, frequently involving both protein calorie and varying degrees of micronutrient deficiency of vitamin A, vitamin E, vitamin B6, folate, zinc, iron, copper, and selenium. PCM leads to atrophy of the lymphoid organs, profound T-lymphocyte deficiency, and increased susceptibility to pathogens, reactivation of viral infections, and development of opportunistic infections [2]. The immune response to infection involves a complex process, including synthesis of acute-phase proteins, cytokines and immunoglobulins and also clonal expansion and cellular differentiation [3]. Clearly this requires an appropriate supply of nutrients to optimize the response and consequently the nutritive status of the host critically determines the outcome of infection.

Effects of nutritional depletion can be found in the innate immune system, for example, lysozyme production by monocytes and polymorphonuclear cells is decreased, complement factors are diminished in both concentration and activity and macrophage functions are also impaired [4]. Multiple abnormalities in specific immunity have also been frequently described in connection with malnutrition. These studies indicate decrease in T-cell function, cytokine production and also in the ability of lymphocytes to respond appropriately to cytokines [5]. T cells have been characterized as Th1 and Th2, depending on their cytokine profile. Th1-type responses are dominated by the production of IFN-γ and are associated with cell-mediated immunity, whereas Th2-type responses are characterized by IL-4 production and more related to humoral responses [6]. In general, innate and cell-mediated immunity are more sensitive to undernutrition than humoral immunity [7]. Nevertheless, more recent investigations also indicate a reduced Th2 activity [8].

Tuberculosis is a disease caused by *Mycobacterium tuberculosis *that is historically known to be particularly influenced by undernutrition. It is a major cause of morbidity and mortality in developing countries where PCM is also prevalent [9]. Even though some reports suggest contribution of humoral immunity against *M. tuberculosis*, it is believe that celular immune response is much more relevant [10-12]. Therefore, the design of all the new vaccines to control TB is based on induction of a predominant cellular immune response. The attenuated BCG strain of *Mycobacterium bovis *has been extensively used as a vaccine against tuberculosis. However, well documented trials showed that the protective efficacy of BCG varies from 0 to 80%. This highly variable and poorly protective efficacy in certain countries has been attributed to the various BCG strains used as vaccines, environmental factors as well as host genetic characteristics [13]. In addition, experimental studies showed that animals were adequately protected by BCG vaccine when properly nourished but exhibited significant weight loss and tuberculin anergy when maintained on a protein-deficient diet [9]. Despite BCG vaccination, malnourished children developed serious and often fatal types of tuberculosis such as miliary, meningitic and disseminated tuberculosis [14].

DNA vaccines represent a promising new approach to vaccination in which the gene for a foreign antigen is expressed within the host's cells. These vaccines generated humoral and cell-mediated immune responses followed by protective efficacy in different experimental models of infectious diseases including tuberculosis. DNA vaccination has been proposed as a hope for better vaccination programs in developing countries [15].

Our group has been working with DNA vaccines constructed by inserting the heat shock protein 65 gene from *Mycobacterium leprae *(hsp65) into plasmid vectors (DNAhsp65). Theoretically, this construction could protect against TB because hsp65 family is one of the most conserved families of proteins presenting more than 97% homology among prokaryotes [16]. In addition, hsp65 and other molecular chaperones are highly immunogenic. Around 10 to 20% of all T cells specifically stimulated are reactive with hsp65 in mice immunized with *M. tuberculosis *[17]. Indeed, this construction displayed both, prophylactic and therapeutic effect in experimental tuberculosis [18,19]. These evaluations were done with mice or guinea pigs submitted to normal chow. Malnutrition could affect both, antigen synthesis and the immune response itself, as they rely on the host's metabolism. Based on this scenario, we hypothesized that immune response induced by a genetic vaccine (pVAXhsp65) could be jeopardized in malnourished mice.

## Materials and methods

### Mice and diets

Isogenic female BALB/c mice, 5–6 weeks old, were housed in plastic cages with white wood chips for bedding and with free access to filtered drinking water, and under controlled conditions of lighting (12 h light/12 h dark cycle) and temperature (23 ± 2°C). After weaning, mice received a 10 day acclimation on a standard chow (Labina, São Paulo, SP, Brazil). This animal chow is considered adequate for mice and is approved by the Brazilian Ministry of Agriculture (n° SP-0311730758). These mice were initially distributed into two groups including a control experimental group (normal), fed *ad libitum *and an undernourished group (restricted) that received 80% of the amount of food consumed by the normal group. Later, they were further allocated to three groups and inoculated with saline solution (vaccine diluent), empty vector (pVAX) or DNA vaccine (pVAXhsp65). Each experimental group included 4 to 8 animals and all evaluations were done at the 40^th ^day after the beginning of dietary restriction.

Animals were manipulated in compliance with the ethical guidelines adopted by the Brazilian College of Animal Experimentation (COBEA), being the experimental protocol approved by the local Ethics Committee.

### Hematological parameters

Blood samples were collected by cardiac puncture and total leukocyte number was counted after blood dilution in Turk's solution. Differential leukocyte count was performed by blood smear stained with eosin/methylene blue (Leishman's stain).

### Histopathological analysis

The whole thymus and a transversal section from small intestine were fixed in formalin (10%), embedded in Paraplast plus (McCormick), prepared routinely and then sectioned for light microscopy. Sections (5 μm each) were stained with haematoxylin and eosin (HE), analyzed in an optical microscope and the images acquired with a digital camera coupled to the microscope.

### Plasmid DNA construction and purification

The vaccine pVAXhsp65 was derived from the pVAX vector that uses the CMV intron (Invitrogen, Carlsbad, CA, USA), previously digested with BamH I and Not I (Gibco BRL, Gaithersburg, MD, USA) to insert a 3.3 kb fragment corresponding to the *M. leprae *hsp65 gene. The empty pVAX vector was used as a control. DH5α *E. coli *transformed with plasmid pVAX or the plasmid carrying the hsp65 gene (pVAXhsp65) were cultured in LB liquid medium (Gibco BRL, Gaithersburg, MD, USA) containing kanamicin (50 μg/ml). The plasmids were purified using the Concert High Purity Maxiprep System (Gibco BRL, Gaithersburg, MD, USA). Plasmid concentrations were determined by spectrophotometry at λ = 260 and 280 nm by using the Gene Quant II apparatus (Pharmacia Biotech, Buckinghamshire, UK).

### Immunization procedures

Normal and restricted groups were immunized by intramuscular route with three doses of pVAXhsp65 (100 μg/100 μl) plus 25% of sucrose (with 10 days interval), being the first dose delivered 10 days after the beginning of dietary restriction. Saline solution or pVAX were also injected in groups submitted to normal or restricted diet.

### Quantification of anti-hsp65 antibodies

Serum samples were obtained by blood centrifugation and anti-hsp65 specific antibody levels were evaluated by enzyme-linked immunosorbent assay (ELISA). Maxisorp plates (Nunc, Life Tech. Inc., USA) were coated with 5 μg/ml of purified recombinant hsp65 in coating solution (Na_2_CO_3_/NaHCO_3_, pH 9.6), at 4°C, overnight. Non-specific protein binding was blocked by incubation with 0.05% Tween 20, 10% fetal calf serum (FCS) in phosphate buffered saline (PBS, 200 μl per well) for 1 h at 37°C. Subsequently, plates were incubated with serum diluted 1:10 (1 h, 37°C). For the detection of specific serum IgG1 and IgG2a, the plates were incubated with biotinylated anti-mouse antibodies (PharMingen, BD Biosciences, USA) for 1 h at 37°C. Plates were then incubated for 30 min at room temperature with Strept AB (kit from Dako, Carpinteria), and revealed by adding H_2_O_2 _with ortho-phenylenediamine (OPD) (Sigma, USA). Color development was stopped with H_2_SO_4 _and optical density was measured at 490 nm.

### Evaluation of cytokine production

Splenic cells were obtained at the 40^th ^day after the beginning of dietary restriction. Cell suspensions were adjusted to 5 × 10^6 ^cells/ml in RPMI 1640 medium, supplemented with 10% FCS, 2 mM L-glutamine and 40 mg/L of gentamicin. The cells were cultured in 48-well flat-bottomed culture plates (Nunc) in the presence of concanavalin A (ConA), 10 μg/ml, type IV-S (Sigma Chemicals, USA), lipopolysaccharide (LPS), 10 μg/ml, *E. coli*, sorotype 055:B5 (Sigma) or fixed *Staphylococcus aureus *Cowan 1 strain (SAC), final diluition 1:2500 (Calbiochem, Behring Co., USA). Cytokine levels were evaluated 48 hours later by ELISA in culture supernatants using anti-IFN-γ and anti-IL-4 as capture antibodies.

### Statistical analysis

Results were expressed as the mean ± SD for each variable. Statistical analysis was performed using Minitab Version 1996 (Minitab Inc, State College, PA, USA). One-way ANOVA and comparative Fisher test were used to analyze the results of antibody production. The other results were analyzed by unpaired t test. Values of p < 0.05 were considered statistically significant.

## Results

### Dietary restriction decreased body and spleen weight

**Figure 1:**
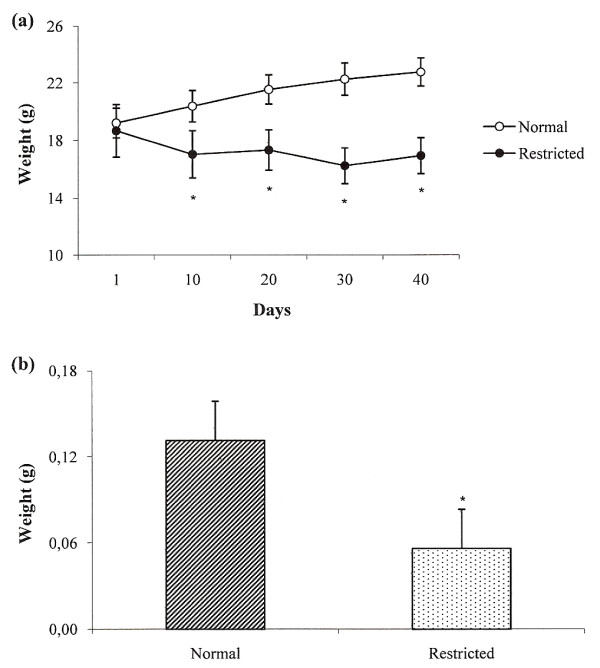
**Effect of dietary restriction on body (a) and spleen (b) weights**. Weight values refer to day 1 (before dietary restriction) and days 10, 20, 30 and 40 after dietary restriction. Spleen weight refers to the 40^th^day of dietary restriction. *Mean value was significantly different from that of the normal group (p < 0.05).

Body weight was daily recorded and losses were already observed 24 h after the beginning of dietary restriction. However, a significant weight loss was detected only from day 4 on. Weight values referring to day 1, before dietary restriction, and days 10, 20, 30 and 40 after dietary restriction are documented in figure 1a. Spleen weight, that was assessed at the 40^th ^day, after animal's euthanasia, was significantly lower in comparison to the control group and it is shown in figure 1b.

### Lymphoid organs were selectively affected during dietary restriction

**Figure 2:**
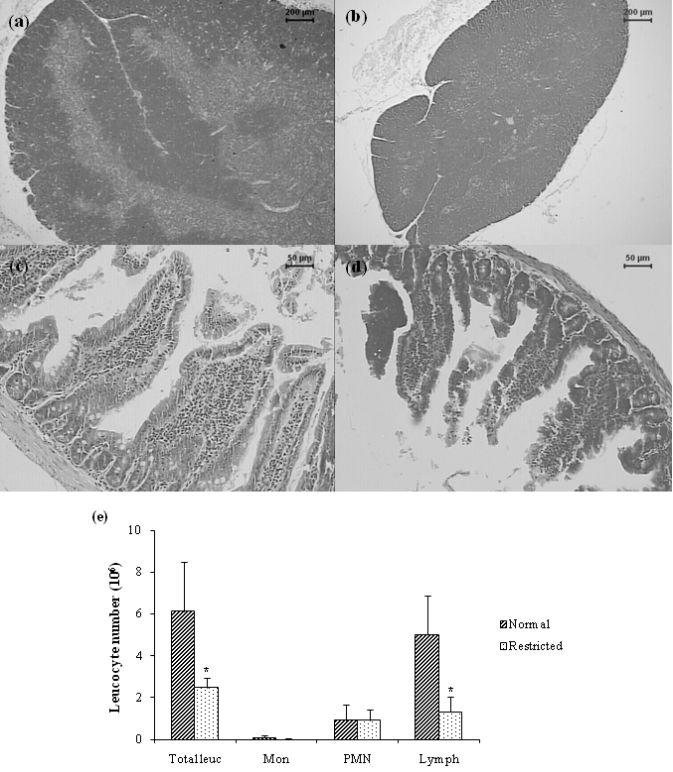
**Effect of dietary restriction on lymphoid organs architecture and on hematological parameters**. Thymus (a, b) and small intestine (c, d) sections stained with HE from BALB/c mice fed with normal diet (left column) or 80% of normal diet (right column). Total and differential number of monocytes, PMN cells and lymphocytes (e). *Mean value was significantly different from that of the normal group (p < 0.05).

By comparison to the normal thymus showed in figure 2a, a severe atrophy is observed in this organ in malnourished animals. Weight evaluation indicated a 52% reduction in comparison to the normal control group (data not shown). In addition to atrophy, the distinction between cortical and medullar areas was also not evident in the group with dietary restriction (figure 2b).

The most striking changes observed in undernourished mice, at the mucous membrane associated with the small intestine, was a villous atrophy. In addition of being smaller and irregular, these intestinal villosities lost their brush borders. Alterations can be observed in figure 2d, comparing to normal structures shown in figure 2c.

### Dietary restriction decreased lymphocytes but not PMN cell number

Total leucocyte number was significantly decreased in undernourished mice comparing to the control group. This reduction coincided with an also significant diminished lymphocyte number. No alteration was detected in the total PMN cell count. These results can be observed in figure 2e.

### Production of IFN-γ and IL-4 was affected by dietary restriction

**Figure 3:**
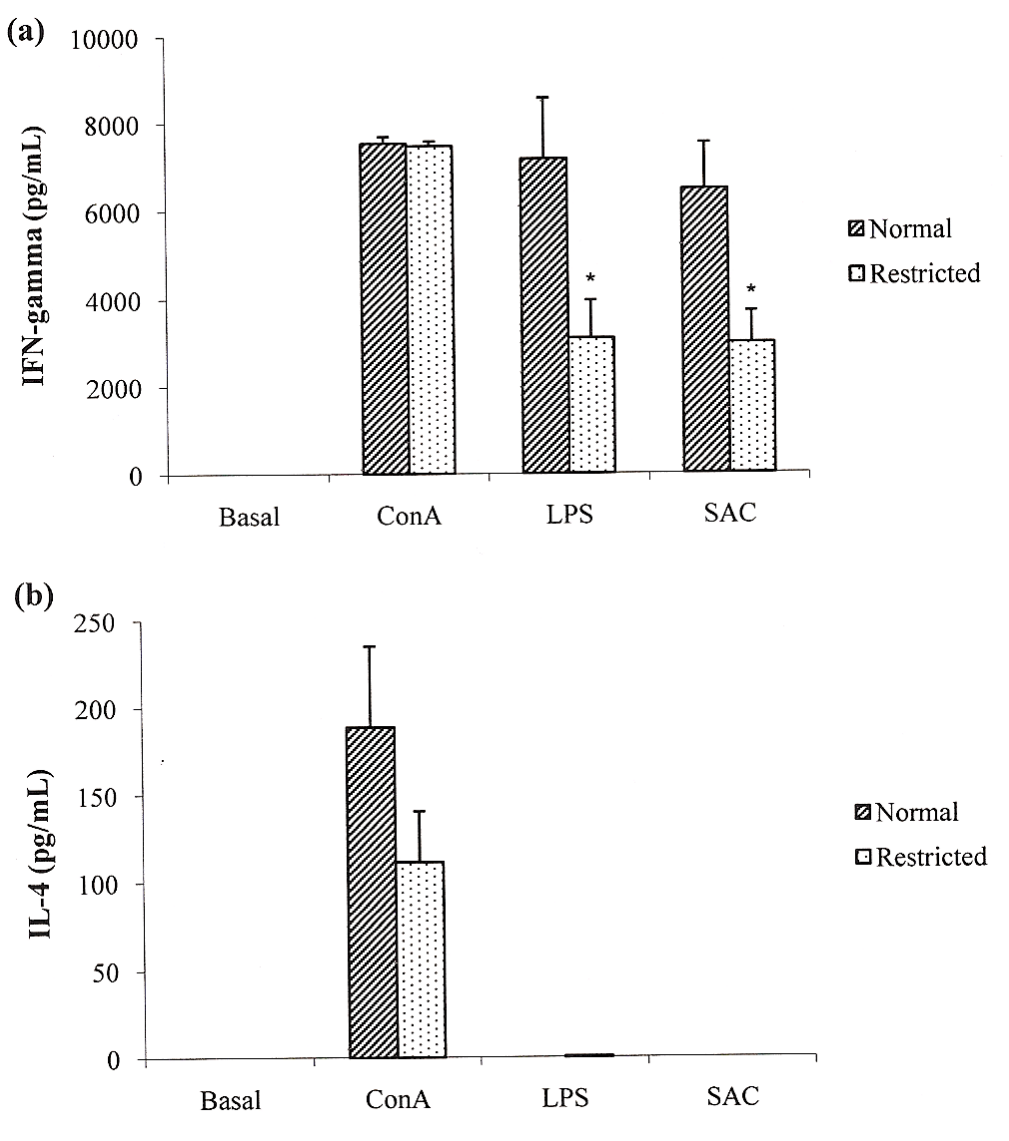
**Effect of dietary restriction on cytokine production by spleen cell cultures**. IFN-γ (a) and IL-4 (b) levels were determined by ELISA in supernatants from cultures stimulated with Concanavalin A (ConA), lipopolysaccharide (LPS) and *S. aureus *(SAC) and non-stimulated cultures (basal). *Mean value was significantly different from that of the normal group (p < 0.05).

Production of IFN-γ, that is documented in figure 3a, varied according to the stimulus. In ConA stimulated cultures there was no difference between control and the experimental group under dietary restriction. However, IFN-γ production was significantly reduced in cultures stimulated with LPS or SAC. IL-4 levels are shown in figure 3b. As can be observed, only ConA addition was able to induce detectable IL-4 levels. The group submitted to dietary restriction showed reduced levels of this cytokine, even though this reduction was not statistically significant.

### Dietary restriction abrogated humoral immune response induced by a DNA vaccine

**Figure 4:**
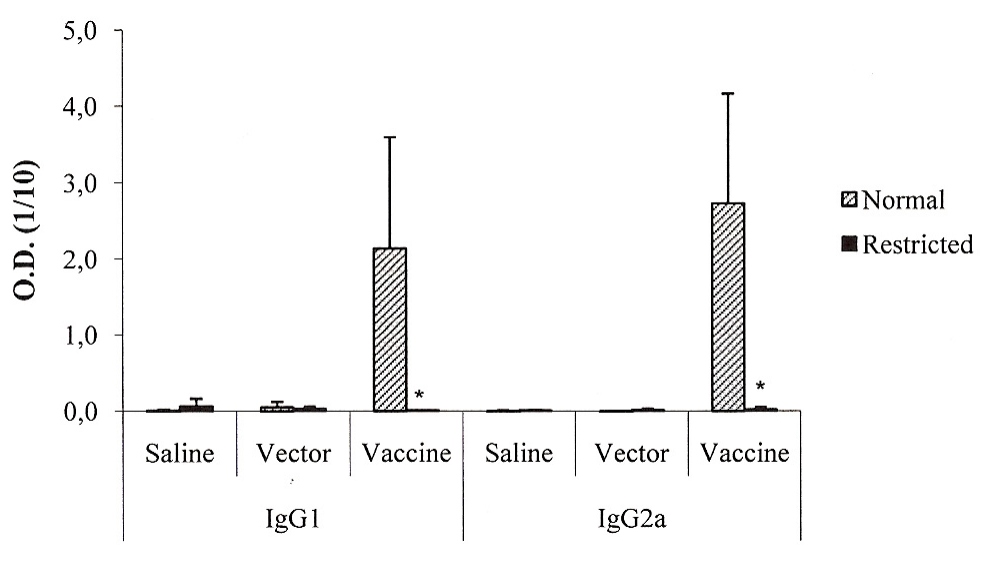
**Effect of dietary restriction on antibody production induced by pVAXhsp65**. Anti-hsp65 antibody production (IgG1 and IgG2a) was tested by ELISA in serum samples from BALB/c mice fed with normal diet (normal) or 80% of normal diet (restricted) groups. *Mean value was significantly different from that of the normal group (p < 0.05).

Immunization of BALB/c mice with pVAXhsp65 vaccine by intramuscular route induced high levels of both, IgG2a and IgG1 specific antibody levels. As expected, no antibodies were induced by inoculation of the empty vector (pVAX). Diet restriction deeply affected the immune response induced by this vaccine, none of these specific isotypes was detected in their serum (figure 4).

## Discussion

Experimental dietary restriction by deprivation of variable percentages of food intake is being used to explore effects of PCM on immunity and susceptibility to infectious agents [20]. In this study, we first characterized the immunological status of mice submitted to a dietary restriction protocol for 40 days and then evaluated the effect of this restriction on their ability to mount an immune response against a DNA vaccine containing the mycobacterial hsp65 gene.

A significant weight loss was already observed at the fourth day of diet and this was maintained until the end of the experiment that was at the 40^th ^day. Weight losses are described in many studies with undernourished animals and used as a criteria to characterize undernutrition. A striking decrease in leucocyte number that selectively affected lymphocytes was also observed.

Alterations in body and spleen weights were compatible with the findings from the histopathological analysis that showed evident alterations in lymphoid organs. Thymus sections from dietary restricted group revealed severe atrophy that was reinforced by a 52% reduction in their weights (not shown). These findings are highly supported by the literature in both, experimental and human malnutrition [21]. Peyer's patches and inguinal lymph nodes were clearly atrophic (not shown). The deleterious effect over mucosal immune system was attested by the evident villous atrophy observed in the small intestine. Sulivan *et al*. [22] have shown that poor dietary protein has a direct effect on mucosal IgA, secretory component, number of IgA-containing cells and IgG levels in rats.

As cytokines are the major effectors and regulators of the immune response, we next evaluated the ability of spleen cells to produce IFN-γ and IL-4 that are considered key cytokines in the development of Th1 and Th2 cells, respectively [23]. As IFN-γ can be directly induced by polyclonal activation of T cells, the spleen cells were stimulated with ConA, LPS and SAC were additionally used because they indirectly induce IFN-γ production by NK cells, i.e, via IL-12 production [24,25]. In ConA stimulated cultures there was no difference between normal and dietary restricted groups. However, IFN-γ production was significantly compromised in cultures stimulated with LPS or *S. aureus *(SAC). This decreased IFN-γ production is consistently described in humans and experimental models with malnutrition [26,27].

The mechanism involved in this differential IFN-γ response associated with distinct stimuli was not investigated. However, we could think that the decreased T cell number was associated with a higher degree of apoptosis as was clearly demonstrated by Pires *et al*. [28]. In this context, the remaining T cells, i.e., the ones spared from apoptosis, could still be able to produce this cytokine if adequately stimulated. This was hypothesized from the additional fact that ConA is a strong stimulus that directly and strongly interacts with glycoproteins from T cell surface [29]. On the other hand, the reduced IFN-γ levels induced by LPS and SAC could indicate that other cell functions or cytokine synthesis are compromised by dietary restriction. IL-12 availability is considered the dominant factor in driving the development of Th1 cells that are characterized by IFN-γ synthesis [30]. Therefore, lower levels of this cytokine could profoundly impair IFN-γ production. It is also well described that IL-12 is involved in IFN-γ production in protocols where LPS and SAC are used to stimulate human cells [31]. The possibility that reduced IFN-γ production is associated with a deficit in IL-12 supply is reinforced by a recent publication in which the authors demonstrated a significant reduction in both, IL-12p70 and IFN-γ synthesis in mice whose diet was reduced to 70% of the amount of food consumed by the corresponding control group [20].

The effect of these alterations on the immune response induced by the pVAXhsp65 vaccine was devastating. In comparison to the control group that produced significant amounts of both, IgG1 and IgG2a anti-hsp65 antibodies, undernourished mice did not produce even basal levels of these antibodies. As Th1 cells are characterized by IFN-γ production and, in mice, the selective switching to IgG2a whereas Th2 cells produce IL-4 and trigger switch to IgG1 and IgE [6] these results indicate that this degree of diet restriction is highly deleterious for both, cellular and humoral components of the immune response.

The effect of the nutritional status during conventional vaccination has been investigated. Measles vaccines did not show efficacy in undernourished children in Africa and India [32]. On the other hand, Moore *et al*. [33] studying the immune response to different vaccines in undernourished children in Gambia, concluded that the secretion of antibodies was not altered even by different degrees of nutritional deficiencies. Only a few reports addressed the consequences of a nutritional deficiency on DNA vaccines. Recently, Sakai *et al*. [34] found a selective impairment of T cells with no effect over B lymphocytes, in a protein deficiency model.

This complete abrogation of the immune response towards a DNA vaccine in undernourished mice could be explained by the double role of the host submitted to this kind of vaccination. In this case, in addition of cellular interactions that are necessary to mount the immune response, the host cells also need to synthesize the antigen. Therefore, it is expected that the immunity to DNA vaccines is even more compromised than the response to conventional vaccines.

Further investigations will be necessary to answer very relevant questions in this area. It will be important to establish if this finding will apply to other plasmids, if other delivery vectors will behave the same way and also if the immunization route can affect the final immune response.

## Conclusion

Together these results demonstrate that a 20% reduction in the amount of food intake was able to significantly alter the immune system. The physiological relevance of these alterations was demonstrated by the abrogation of the immune response induced by a DNA vaccine against tuberculosis. These results alert for the fundamental role of the nutritional state, which is frequently affected in developing countries, in vaccine programs.

## Competing interests

The authors declare that they have no competing interests.

## Authors' contributions

LLWI, TGDF and AS are the main investigators in this study. FCM, SFGZP and NMM largely contributed with the immunological experiments. PCMP and CLS provided critical input.
